# Two-Dimensional Cephalometric Assessment of Skeletal and Dentoalveolar Changes Following Alt-RAMEC with a 2-Hinged Maxillary Expander and RME/Facemask Therapy in Skeletal Class III Patients with Maxillary Retrusion: A Retrospective Comparative Study

**DOI:** 10.3390/diagnostics16162630

**Published:** 2026-08-19

**Authors:** Nuri Can Tanrısever, Hatice Gökalp

**Affiliations:** Department of Orthodontics, School of Dentistry, University of Ankara, Ankara 06560, Türkiye

**Keywords:** retrognathia, maxilla, palatal expansion technique, cephalometry, orthodontic appliance design, osteotomy, Le Fort, Alt-RAMEC, 2-hinged maxillary expander

## Abstract

**Background/Objectives:** This retrospective cephalometric study evaluated skeletal, alveolar, and dentoalveolar changes following alternate rapid maxillary expansion and constriction using a two-hinged maxillary expander (Alt-RAMEC/2-HME) and rapid maxillary expansion with facemask therapy (RME/FM) in skeletal Class III patients with maxillary retrusion, using a Le Fort I osteotomy group as a surgical reference. **Methods:** Pre-treatment (T0) and post-treatment (T1) lateral cephalometric radiographs of 42 patients were analyzed. Patients were divided into three groups—Alt-RAMEC/2-HME (n = 14), RME/FM (n = 14), and Le Fort I osteotomy (n = 14)—representing late-growth adolescents, growing children, and mature adults, respectively. Sagittal and vertical landmark positions were measured using a Cartesian coordinate system. The primary outcome was sagittal A-point displacement between T0 and T1. Intergroup changes were compared using non-parametric tests (*p* < 0.05). **Results:** Sagittal A-point displacement increased significantly in all groups. A larger A-point displacement was observed in the Le Fort I osteotomy group than in the two orthopedic groups (*p* < 0.001), whereas no significant difference was observed between the Alt-RAMEC/2-HME and RME/FM groups. Greater dentoalveolar positional changes were observed in the RME/FM group, in the maxillary incisor and molar regions. **Conclusions:** Sagittal A-point displacement was observed following Alt-RAMEC/2-HME in late-growth skeletal Class III patients with maxillary retrusion. However, in the absence of an untreated age- and skeletal maturity-matched control group, the observed displacement cannot be distinguished definitively from the normal craniofacial growth trajectory. These findings should also be interpreted in light of the retrospective study design and baseline differences between groups.

## 1. Introduction

Class III malocclusion associated with maxillary retrusion is a clinically relevant dentofacial problem in growing individuals and may negatively affect facial esthetics, occlusal function, and psychosocial well-being if left untreated. In Class III malocclusion characterized by maxillary retrusion, with or without transverse deficiency, maxillary expansion has been used not only to correct transverse discrepancies but also to influence the sutural structures directly or indirectly connected to the maxilla [1]. Palatal expansion may affect not only the midpalatal suture but also the circumzygomatic and circummaxillary sutural systems [2].

Because the skeletal response to maxillary expansion depends on the distribution of orthopedic forces throughout the craniofacial complex, previous experimental studies using dry skulls, rhesus monkeys, and finite element models have investigated the biological and biomechanical effects of expansion protocols [3,4,5,6,7,8,9,10,11]. The maxilla articulates with adjacent craniofacial bones through multiple sutures, allowing expansion forces to be transmitted both within the maxilla and to surrounding circummaxillary structures [4,9]. However, the response of the circummaxillary sutural system to rapid maxillary expansion (RME) may be limited by the heavy interdigitation of the sutures around the palatine bone. In particular, coronally oriented circummaxillary sutures have been reported to open less than sagittally oriented sutures [12].

The magnitude and direction of the skeletal response may be influenced by several factors, including appliance design, activation protocol, force distribution, and sutural orientation [12,13,14,15,16,17]. In this context, Liou and Tsai introduced the 2-Hinged Maxillary Expander (2-HME) in combination with the Alternate Rapid Maxillary Expansion and Constriction (Alt-RAMEC) protocol. This approach was proposed to enhance circummaxillary sutural disarticulation and promote sagittal maxillary displacement while limiting excessive transverse expansion [18,19]. In conventional Hyrax-type expansion, the center of rotation has been described near the posterior nasal spine, which may contribute to posterior rotation of the maxilla. In contrast, the 2-HME design has been suggested to shift the center of rotation toward the pterygoid region, potentially affecting the direction of maxillary displacement [18,19].

RME followed by facemask (FM) therapy is a widely used orthopedic approach for growing Class III patients with maxillary retrusion [13,14,15,20,21,22,23]. However, when skeletal growth approaches completion, the orthopedic treatment response becomes less predictable, and the distinction between remaining orthopedic potential and the need for surgical correction becomes clinically important. After growth completion, Le Fort I osteotomy remains the established surgical approach for correcting maxillary deficiency [24].

Although Alt-RAMEC protocols have been reported to facilitate anterior maxillary displacement in growing patients, the cephalometric pattern of skeletal, alveolar, and dentoalveolar changes produced by Alt-RAMEC/2-HME in patients approaching skeletal maturity remains insufficiently clarified [25]. Moreover, limited evidence is available regarding how sagittal A-point displacement after Alt-RAMEC/2-HME compares with that observed after conventional RME/FM therapy when assessed using a standardized cephalometric coordinate system. Including a Le Fort I osteotomy group as a surgical reference may help contextualize the magnitude of orthopedic maxillary displacement, rather than serving as a directly comparable treatment modality.

Therefore, the aim of this retrospective cephalometric study was to evaluate skeletal, alveolar, and dentoalveolar positional changes following Alt-RAMEC/2-HME and conventional RME/FM therapy in skeletal Class III patients with maxillary retrusion, using Le Fort I osteotomy as a surgical reference for maxillary skeletal displacement. The primary outcome was sagittal A-point displacement between T0 and T1. The null hypothesis was that there would be no significant difference in sagittal A-point displacement between the Alt-RAMEC/2-HME and conventional RME/FM groups.

## 2. Materials and Methods

### 2.1. Study Design and Ethical Approval

This single-center retrospective comparative study was conducted using archived lateral cephalometric radiographs obtained from the Department of Orthodontics, Faculty of Dentistry, Ankara University. The study was designed to evaluate treatment-related skeletal and dentoalveolar positional changes using a standardized two-dimensional cephalometric coordinate system. The study protocol was approved by the Ankara University Faculty of Dentistry Ethics Committee (Meeting No: 4; Decision Date: 23 December 2024) and was conducted in accordance with the principles of the Declaration of Helsinki.

### 2.2. Sample Size Calculation

A priori sample size calculation was performed using G*Power version 3.1. The calculation was based on the primary outcome variable, defined as the treatment-related sagittal displacement of A-point (ΔA-Y). The selected test family was the F tests, and the selected statistical test was ANOVA: repeated measures, within-between interaction, with three groups and two repeated measurements. In the absence of directly comparable previous data for this specific three-group comparison, a large effect size was assumed for sample size estimation. The input parameters were as follows: effect size f = 0.50, alpha error probability = 0.05, statistical power = 0.90, number of groups = 3, number of measurements = 2, allocation ratio = 1:1:1, correlation among repeated measures = 0.50, and nonsphericity correction ε = 1. Based on these parameters, the minimum required sample size was calculated as 39 individuals, with at least 13 participants per group. Therefore, 42 individuals were included, with 14 participants in each group.

### 2.3. Participants and Eligibility Criteria

This retrospective study was conducted using archived clinical and radiographic records of patients treated between January 2014 and September 2024. A total of 96 archived patient records were initially screened for eligibility. Eligibility screening and the application of the inclusion and exclusion criteria were performed by the same investigator (N.C.T.) who subsequently conducted the cephalometric measurements.

Participants were included if they met the following criteria:

1. Skeletal Class III malocclusion primarily associated with maxillary retrusion, diagnosed on lateral cephalometric radiographs as an ANB angle <0° together with an SNA angle <80°. These cutoff values were used to ensure that the Class III skeletal pattern was primarily related to maxillary retrusion rather than isolated mandibular prognathism.
2. Age criteria appropriate to the treatment modality: 14–17 years with advanced but incomplete skeletal growth for the Alt-RAMEC/2-HME group, 9–13 years for the RME/FM group, and ≥18 years with skeletal maturity for the Le Fort I osteotomy group.
3. No previous history of orthodontic, orthopedic, or orthognathic treatment before the evaluated treatment protocol.
4. Availability of high-quality pre-treatment (T0) and post-treatment (T1) lateral cephalometric radiographs suitable for cephalometric analysis.
5. Availability of hand-wrist radiographs for skeletal maturation assessment in the orthopedic groups.
6. Completion of the planned orthopedic or surgical treatment protocol and availability of complete clinical records.

Patients were excluded if they had:

1. Craniofacial syndromes or congenital anomalies, such as cleft lip and palate;
2. History of facial trauma or previous orthognathic surgery;
3. Systemic diseases affecting skeletal growth or bone metabolism;
4. Severe facial asymmetry requiring additional surgical intervention;
5. Incomplete clinical records or radiographs unsuitable for cephalometric analysis.

After applying these eligibility criteria, 17 patients were excluded because they did not meet the cephalometric criteria for skeletal Class III malocclusion primarily associated with maxillary retrusion, 12 patients were excluded because of missing or poor-quality T0 or T1 lateral cephalometric radiographs, 9 patients were excluded because of previous orthodontic, orthopedic, or orthognathic treatment, 10 patients were excluded because they did not meet the age or skeletal maturation criteria for the relevant treatment group, and 6 patients were excluded because of craniofacial anomalies, severe facial asymmetry, systemic conditions affecting growth, or incomplete clinical records. Finally, 42 individuals met the inclusion criteria and were included in the study.

The final study sample consisted of pre-treatment (T0) and post-treatment (T1) lateral cephalometric radiographs of 42 individuals with skeletal Class III malocclusion primarily associated with maxillary retrusion. Participants were classified into three groups according to the treatment modality recorded in the clinical archive.

Group 1 (Alt-RAMEC/2-HME) included 14 patients treated with the alternate rapid maxillary expansion and constriction protocol using a two-hinged maxillary expander (8 females, 6 males; mean age: 15.35 ± 1.01 years; mean skeletal age: 15.25 ± 1.17 years).

Group 2 (RME/FM) included 14 patients treated with conventional rapid maxillary expansion followed by facemask therapy (7 females, 7 males; mean age: 11.40 ± 1.16 years; mean skeletal age: 12.25 ± 1.02 years).

Group 3 (Le Fort I osteotomy) included 14 adult patients who underwent surgical maxillary advancement and served as a surgical reference group for skeletal maxillary displacement (6 females, 8 males; mean age: 24.75 ± 1.57 years).

Skeletal maturation was assessed in the orthopedic groups using hand–wrist radiographs according to the Greulich and Pyle Atlas method. The Le Fort I osteotomy group consisted of skeletally mature adult patients; therefore, hand–wrist maturation assessment was not required for this group.

Because the treatment modalities represented different clinical indications, baseline age and skeletal maturation differences among groups were expected. Conventional RME/FM therapy was performed in younger growing patients, whereas the Alt-RAMEC/2-HME protocol was applied to patients in the late-growth period. The Le Fort I osteotomy group was included as an adult surgical reference group rather than as an age-matched orthopedic comparison group.

### 2.4. Treatment Protocols

**Figure 1:**
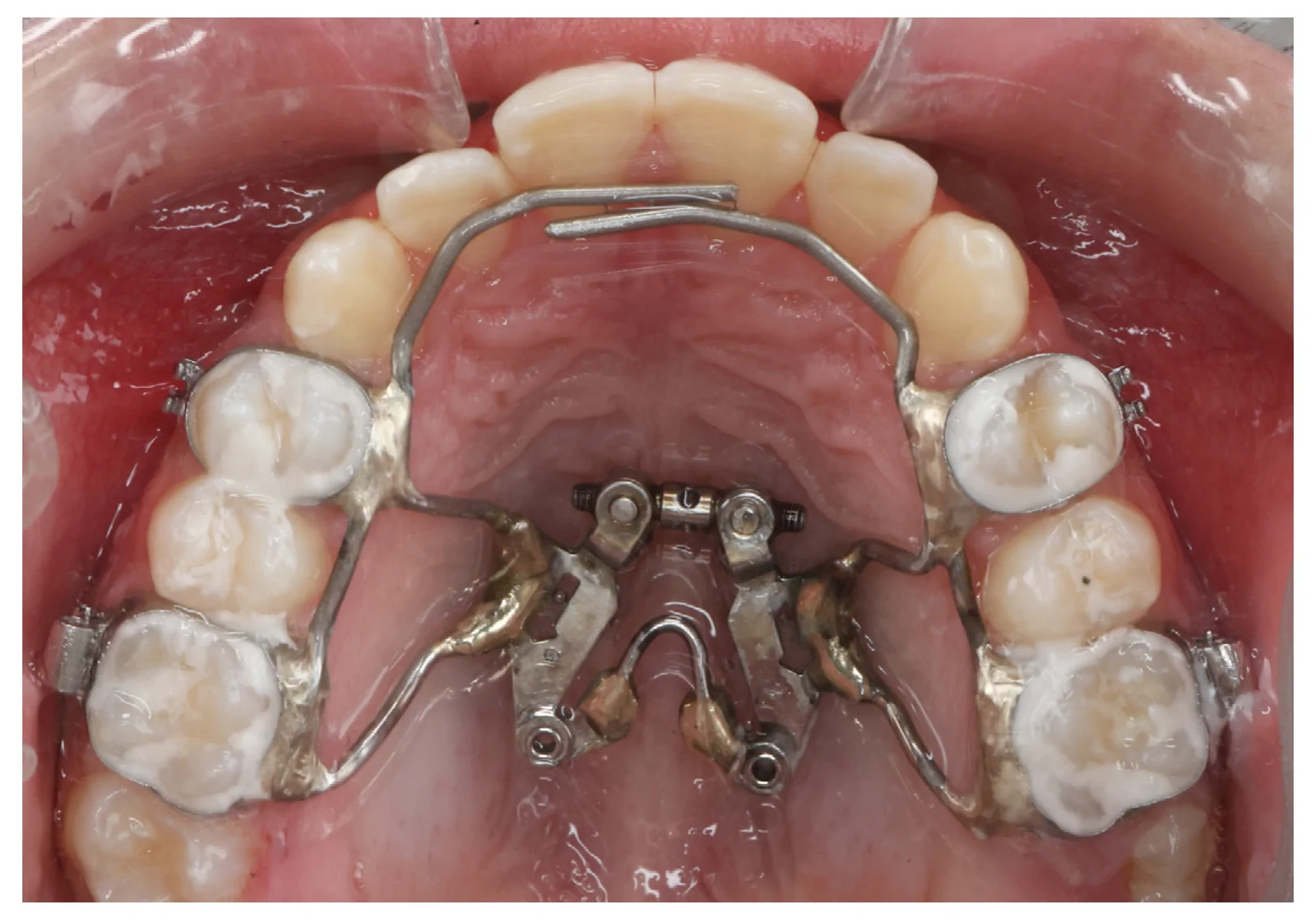
Design of the two-hinged maxillary expander (2-HME) used in the Alt-RAMEC protocol. The intraoral photograph is from a study participant and was obtained from the archived clinical records. The appliance consists of two posterior hinges positioned near the maxillary molar bands and an expansion screw placed parallel to the occlusal plane.

In Group 1, the 2-Hinged Maxillary Expander (2-HME), shown in Figure 1, was activated four quarter-turns per day, corresponding to 1 mm of nominal screw activation per day.

During each expansion week, the screw was activated for 7 consecutive days, resulting in 28 quarter-turns and 7 mm of expansion activation. During the following constriction week, the screw was reversed using the same activation rhythm, corresponding to 28 reverse quarter-turns and 7 mm of constriction activation. This alternating expansion–constriction sequence was repeated for 9 consecutive weeks and ended with an expansion phase. Therefore, the protocol consisted of five expansion weeks and four constriction weeks, resulting in a cumulative nominal screw activation of 35 mm in the expansion direction and 28 mm in the constriction direction, with a final net expansion activation of 7 mm.

In Group 2, a McNamara-type Hyrax expander was activated twice daily, once in the morning and once in the evening, producing 0.5 mm of expansion per day for one week using a conventional rapid maxillary expansion protocol. Following completion of active expansion, a Petit-type facemask was used with bilateral 450-g elastics extending from the premolar region of the expander in a downward and forward direction, below the maxillary occlusal plane, at approximately 40° to the maxillary occlusal plane. Patients were instructed to wear the facemask for approximately 21 h per day. The total treatment duration was 10.1 ± 1.9 months.

In Group 3, maxillary advancement was performed using Le Fort I osteotomy as part of the surgical correction of skeletal Class III maxillary deficiency.

For standardized cephalometric endpoint assessment, post-treatment radiographs (T1) were selected according to the protocol-specific endpoint of each group rather than after a standardized observation interval. Accordingly, T1 records were obtained after completion of the 9-week Alt-RAMEC/2-HME protocol in Group 1, after a mean treatment duration of 10.1 ± 1.9 months following completion of the orthopedic protraction phase in Group 2, and immediately after Le Fort I osteotomy in Group 3. Consequently, treatment-related changes reflected protocol-specific clinical endpoints rather than rates of skeletal change over equivalent follow-up periods.

### 2.5. Cephalometric Analysis

**Figure 2:**
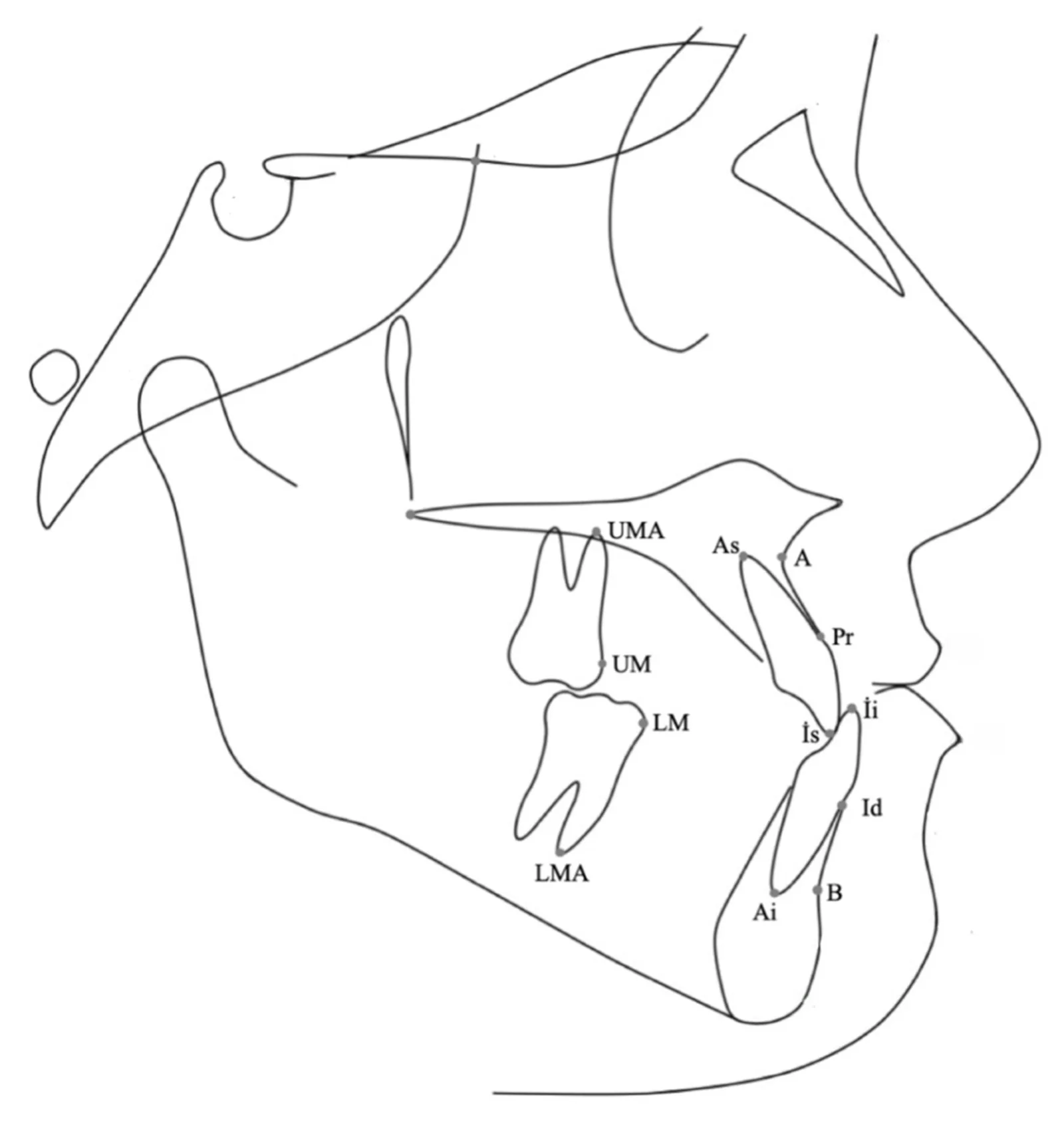
Cephalometric landmarks used for sagittal and vertical linear measurements. A, subspinale (A-point); B, supramentale (B-point); Is upper incisor edge; As, upper incisor apex; Ii, lower incisor edge; Ai, lower incisor apex; Pr, prosthion; Id, infradentale; UM, most mesial point of the crown contour of the maxillary first molar; UMA, apex of the mesiobuccal root of the maxillary first molar; LM, most mesial point of the crown contour of the mandibular first molar; LMA, apex of the mesial root of the mandibular first molar.

**Figure 3:**
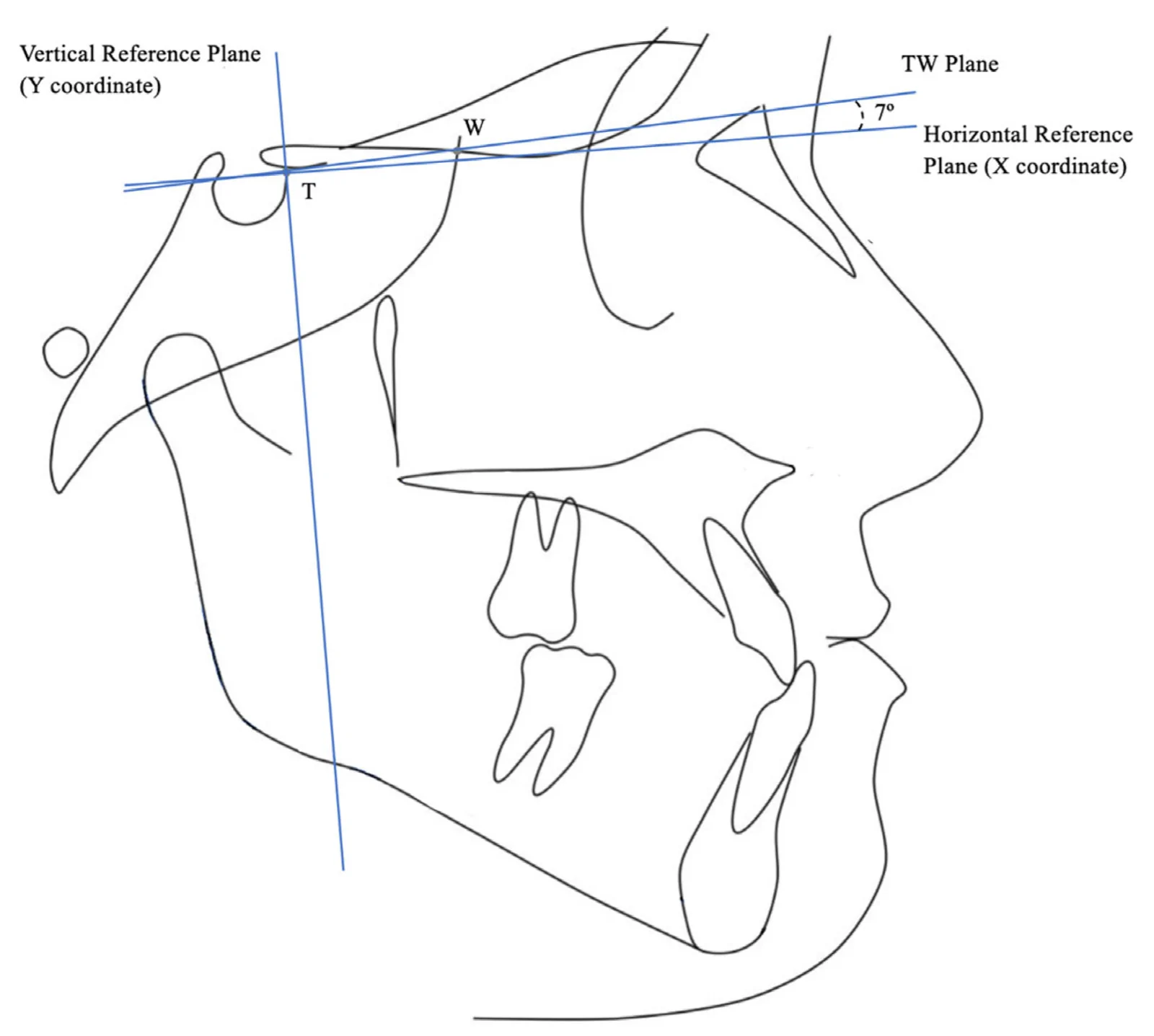
Cartesian coordinate system used for cephalometric measurements. The horizontal reference plane (*X*-axis) was constructed at an angle of +7° relative to the TW plane passing through the Tuberculum Sella (T) and Wings (W) points. The vertical reference plane (*Y*-axis) was constructed perpendicular to the *X*-axis through the T point.

Sagittal and vertical linear skeletal and dental measurements were performed on lateral cephalometric radiographs using a Cartesian coordinate system and predefined cephalometric landmarks (Figure 2 and Figure 3). All radiographs were calibrated using the 10-mm cephalostat reference bar visible on the digital images.

The Cartesian coordinate system was constructed using the TW plane passing through the Tuberculum Sella (T) and Wings (W) points as the cranial reference plane. The *X*-axis was constructed from the T point at an angle of +7° relative to the TW plane. The *Y*-axis was constructed perpendicular to the *X*-axis through the T point (Figure 3). Sagittal positions were recorded as linear distances measured relative to the *Y*-axis, and vertical positions were recorded as linear distances measured relative to the *X*-axis. Accordingly, A-Y represented the sagittal position of A-point, whereas A-X represented the vertical position of A-point. An increase in A-Y was interpreted as anterior displacement of A-point.

All cephalometric measurements were performed by the same investigator (N.C.T.) using AutoCAD software (AutoCAD 2025, Autodesk, San Francisco, CA, USA). Linear measurements were recorded in millimeters.

### 2.6. Study Outcomes

The primary outcome measure was defined a priori as the sagittal displacement of A-point between T0 and T1 (ΔA-Y), representing the sagittal positional change of the maxillary skeletal reference point. Secondary skeletal and alveolar outcomes included sagittal and vertical positional changes of B-point, prosthion, and infradentale, as well as the vertical positional change of A-point. Dentoalveolar positional changes of the maxillary and mandibular incisors and first molars were considered secondary dentoalveolar outcomes. These additional measurements were analyzed to characterize the skeletal, alveolar, and dentoalveolar pattern of treatment response and were interpreted as supportive findings.

### 2.7. Reliability Assessment

To assess intra-observer reliability, all cephalometric measurements were repeated by the same investigator after a one-week interval. Because of the retrospective nature of the study, the investigator was not blinded to the treatment group or timepoint during the measurements. Intraclass correlation coefficients were calculated using a two-way mixed-effects model, absolute agreement definition, and single-measures approach [ICC(3,1)]. ICC values greater than 0.90 were considered to indicate excellent reliability.

### 2.8. Statistical Analysis

Statistical analyses were performed using IBM SPSS Statistics version 26.0. The data distribution was assessed using the Shapiro–Wilk test. Because the assumption of normality was not met for the main study variables, non-parametric tests were used. Continuous variables are summarized as mean ± standard deviation, and categorical variables are summarized as frequencies and percentages.

Baseline demographic and skeletal characteristics were compared among the study groups. Continuous variables were compared using the Kruskal–Wallis test. Skeletal age was compared only between the two orthopedic groups using the Mann–Whitney U test because hand–wrist maturation assessment was not performed in the skeletally mature Le Fort I osteotomy group. Categorical variables were compared using the chi-square test or Fisher’s exact test, as appropriate.

Within-group comparisons between T0 and T1 were performed using the Wilcoxon signed-rank test. Intergroup comparisons of treatment-related changes (T1–T0) among the three groups were performed using the Kruskal–Wallis test. The reported Kruskal–Wallis *p*-values represent the overall (omnibus) intergroup comparisons and were not adjusted for multiple testing. When significant intergroup differences were detected, Bonferroni-adjusted pairwise comparisons were performed using the Mann–Whitney U test. Although all three groups were included in the intergroup analyses, the Le Fort I osteotomy group was included as a quantitative surgical reference to contextualize the magnitude of maxillary skeletal displacement rather than as an age- and indication-matched therapeutic comparator. Because the study groups represented different clinical indications and differed substantially in age, skeletal maturation, baseline sagittal skeletal characteristics, and observation period, the intergroup analyses were intended to provide quantitative comparisons of the observed positional changes rather than to establish treatment superiority or attribute the observed differences solely to the treatment modalities.

The primary outcome was ΔA-Y. Analyses of secondary skeletal, alveolar, and dentoalveolar outcomes were considered exploratory. Therefore, no adjustment was applied for multiplicity across the full set of secondary endpoints, and these findings should be interpreted cautiously rather than as confirmatory evidence. Statistical significance was set at *p* < 0.05.

## 3. Results

The baseline demographic and skeletal characteristics of the study groups are summarized in Table 1. Significant intergroup differences were observed in age, SNA, and ANB values, whereas sex distribution and SNB values did not differ significantly among the groups. Skeletal age was compared only between the two orthopedic groups because hand–wrist maturation assessment was not performed in the skeletally mature Le Fort I osteotomy group, and a significant difference was observed between these two groups.

Intra-observer reliability was excellent for all cephalometric measurements, with ICC(3,1) values ranging from 0.912 to 0.987, indicating a high level of measurement consistency.

For the primary outcome, A-Y showed a significant increase between T0 and T1 in all three groups (*p* < 0.05) (Table 2). Regarding secondary skeletal and alveolar outcomes, Pr-Y showed significant changes in all three groups, whereas Id-Y showed significant changes only in the two orthopedic groups. B-Y showed a significant change only in the RME/FM group. In vertical measurements, A-X did not show a significant change in any group. B-X showed significant changes in both orthopedic groups, Pr-X showed significant changes in the RME/FM and Le Fort I osteotomy groups, and Id-X showed significant changes in all three groups. The corresponding mean treatment-related changes (Δ = T1 − T0) together with their 95% confidence intervals are presented in Supplementary Table S1.

For secondary dentoalveolar outcomes, sagittal positional changes of the maxillary incisor and maxillary first molar landmarks were significant in all three groups (Table 3). Lower incisor edge position (II-Y) showed significant changes in both orthopedic groups, whereas lower incisor apex position (AI-Y) showed a significant change only in the Le Fort I osteotomy group. Lower molar crown position (LM-Y) showed a significant change only in the Alt-RAMEC/2-HME group, while lower molar root apex position (LMA-Y) did not show a significant change in any group.

In vertical dentoalveolar measurements, significant changes varied among the groups. In the Alt-RAMEC/2-HME group, significant changes were observed in AI-X, UM-X, and LMA-X. In the RME/FM group, significant changes were observed in IS-X, II-X, AI-X, UM-X, UMA-X, LM-X, and LMA-X. In the Le Fort I osteotomy group, significant changes were observed in AS-X, II-X, AI-X, UM-X, UMA-X, and LM-X. The corresponding mean treatment-related changes (Δ = T1 − T0) together with their 95% confidence intervals are presented in Supplementary Table S2.

An intergroup comparison of treatment-related skeletal and alveolar linear changes is presented in Table 4. For the primary outcome, ΔA-Y differed significantly among the three groups (*p* < 0.001). Bonferroni-adjusted pairwise comparisons showed that the Le Fort I osteotomy group had significantly greater ΔA-Y than both orthopedic groups, whereas no statistically significant difference was observed between the Alt-RAMEC/2-HME and RME/FM groups.

Among the secondary skeletal and alveolar outcomes, Pr-Y also differed significantly among the three groups, with the largest change observed in the Le Fort I osteotomy group, followed by the RME/FM and Alt-RAMEC/2-HME groups. No significant intergroup differences were observed for B-Y, Id-Y, or A-X. In vertical measurements, B-X and Id-X showed significantly greater changes in the two orthopedic groups than in the Le Fort I osteotomy group, while Pr-X showed a significant difference only between the RME/FM and Le Fort I osteotomy groups.

An intergroup comparison of treatment-related dentoalveolar linear changes is presented in Table 5. In sagittal dentoalveolar measurements, significant intergroup differences were observed for IS-Y, AS-Y, II-Y, UM-Y, and UMA-Y. The Le Fort I osteotomy group showed the largest sagittal changes in the maxillary incisor and maxillary first molar landmarks. Among the two orthopedic groups, the RME/FM group showed greater sagittal changes in the maxillary incisor edge and maxillary first molar landmarks than the Alt-RAMEC/2-HME group. No significant intergroup differences were observed for AI-Y, LM-Y, or LMA-Y.

In vertical dentoalveolar measurements, significant intergroup differences were observed for II-X, AI-X, UM-X, UMA-X, LM-X, and LMA-X. For II-X, AI-X, UM-X, LM-X, and LMA-X, pairwise comparisons showed significantly greater changes in the two orthopedic groups than in the Le Fort I osteotomy group. For UMA-X, the RME/FM group showed a significantly greater change than both the Alt-RAMEC/2-HME and Le Fort I osteotomy groups. No significant intergroup differences were observed for IS-X or AS-X.

## 4. Discussion

The present retrospective cephalometric study evaluated skeletal, alveolar, and dentoalveolar positional changes following Alt-RAMEC/2-HME and conventional RME/FM therapy in skeletal Class III patients with maxillary retrusion, including a Le Fort I osteotomy group as an adult surgical reference group for maxillary skeletal displacement. The main finding was that sagittal A-point displacement increased significantly in all three groups. The Le Fort I osteotomy group showed significantly greater sagittal A-point displacement than both orthopedic groups, whereas no statistically significant difference was observed between the Alt-RAMEC/2-HME and conventional RME/FM groups. Therefore, the null hypothesis that there would be no significant difference in sagittal A-point displacement between the two orthopedic groups could not be rejected.

The absence of a statistically significant difference between the Alt-RAMEC/2-HME and RME/FM groups should be interpreted cautiously because the two orthopedic groups differed not only in age, skeletal maturation, and baseline sagittal skeletal characteristics (SNA and ANB), but also in the duration of the observation period. As post-treatment radiographs were obtained at protocol-specific clinical endpoints, the observed positional changes reflect the overall treatment response achieved at the completion of each protocol. Consequently, differences in baseline skeletal severity and observation period may also have contributed to the magnitude of the observed treatment-related positional changes. Under these circumstances, it is difficult to determine to what extent the observed intergroup differences reflect the treatment protocols themselves rather than differences in baseline patient characteristics.

The observed sagittal A-point changes should also be interpreted in the context of untreated Class III growth. A previous study reported approximately 0.2 mm of anterior A-point displacement over a 6-month period in untreated Class III subjects, indicating that physiological growth may produce a small but measurable sagittal change in A-point position [26]. In addition, untreated Class III subjects demonstrate continued increases in midfacial length during growth, including the adolescent period, although increases in midfacial length do not necessarily translate directly into equivalent anterior displacement of A-point [27]. Considering the substantially longer observation period in the RME/FM group, spontaneous growth may therefore have contributed to part of the observed ΔA-Y in this group. In contrast, the relatively short 9-week observation period in the Alt-RAMEC/2-HME group suggests that the growth-related contribution was likely more limited. Assuming a linear rate of change based on the previously reported value of approximately 0.2 mm over 6 months, this rate would correspond to approximately 0.34 mm over the mean 10.1-month observation period in the RME/FM group and approximately 0.07 mm over the 9-week observation period in the Alt-RAMEC/2-HME group. These estimates correspond to approximately 12.5% and 3.7%, respectively, of the observed mean ΔA-Y values in the two groups. However, these proportions should be regarded only as approximate contextual estimates because they are derived from external longitudinal data and assume a linear growth pattern. Moreover, because the cited studies used different age ranges and cephalometric reference systems, and because no untreated age- and skeletal maturity-matched control group was available, the relative contributions of physiological growth and treatment cannot be determined precisely.

The observed sagittal A-point displacement after Alt-RAMEC/2-HME may be related to the biomechanical characteristics of the alternating expansion–constriction protocol and the two-hinged expander design. The 2-HME has been suggested to shift the center of rotation toward the palatomaxillary and pterygomaxillary regions rather than the posterior nasal spine, as described for conventional Hyrax-type expansion. This proposed mechanism may influence the direction of maxillary displacement by modifying the distribution of forces across the circummaxillary sutural system [18,19]. Liou and Tsai also suggested that repeated expansion–constriction cycles may induce a distraction osteogenesis-like biological response in the circummaxillary sutures, potentially facilitating anterior maxillary displacement [18]. Although the sagittal A-point changes observed in the present study are compatible with this proposed biological rationale, the underlying sutural mechanism was not directly evaluated and therefore cannot be confirmed by the present two-dimensional cephalometric findings.

Conventional rapid maxillary expansion followed by facemask therapy remains a widely used orthopedic protocol for the treatment of skeletal Class III malocclusion associated with maxillary retrusion [20,28]. Previous studies have reported anterior maxillary displacement after Alt-RAMEC combined with facemask therapy [18,29,30,31] and after RME/FM therapy [32,33] in growing patients. In the present study, the mean sagittal A-point displacement was numerically smaller in the Alt-RAMEC/2-HME group than in the RME/FM group (1.87 mm vs. 2.69 mm), although this difference was not statistically significant. This finding may be partly related to the more advanced skeletal maturation and shorter observation period of the Alt-RAMEC/2-HME group. In addition, individual variation in skeletal maturation, including potential sex-related differences in maturation timing, should also be considered when interpreting these findings. Accordingly, the observed numerical difference between the two orthopedic groups should be interpreted cautiously, as it may reflect differences in patient characteristics rather than the effects of the treatment protocols alone.

A secondary finding of this study was the difference in dentoalveolar positional changes between the two orthopedic groups. The RME/FM group showed greater sagittal changes in the maxillary incisor edge and maxillary first molar landmarks than the Alt-RAMEC/2-HME group, whereas relatively smaller dentoalveolar positional changes were observed in the Alt-RAMEC/2-HME group. Because the study groups differed substantially in baseline characteristics and observation period, these findings should be interpreted with caution. However, because the present study was based on two-dimensional cephalometric measurements, the relative skeletal and dental contributions to the observed positional changes cannot be quantified precisely. Accordingly, the observed positional changes in dentoalveolar landmarks may represent a combination of tooth movement and displacement of the alveolar process rather than isolated dental movement.

Vertical dentoalveolar changes also differed between the groups. In the RME/FM group, significant vertical changes were observed in several maxillary and mandibular dental landmarks. These findings may be partly associated with the direction of facemask traction, which was applied downward and forward at approximately 40° to the maxillary occlusal plane. Such force application may contribute to the observed vertical positional changes. Nevertheless, these interpretations should remain cautious because mandibular rotation and three-dimensional dental movements cannot be fully characterized using lateral cephalometric radiographs alone.

As expected for a surgical advancement procedure, a larger sagittal A-point displacement was observed in the Le Fort I osteotomy group than in the two orthopedic groups. This finding is consistent with the role of Le Fort I osteotomy in correcting maxillary deficiency after growth completion [24]. The magnitude of maxillary advancement observed in the surgical group was also consistent with previous reports describing approximately 5–8 mm of advancement following Le Fort I osteotomy [34]. Although the Le Fort I group was included in the intergroup statistical analyses, it was intended solely as a quantitative surgical reference to contextualize the magnitude of maxillary skeletal displacement observed after the orthopedic protocols, rather than as an age- and indication-matched therapeutic comparator.

Although surgical correction remains the established approach for maxillary deficiency after growth completion, it may be associated with intraoperative and postoperative complications, including vascular injury, neurosensory disturbances, skeletal relapse, and postoperative stability concerns [35,36]. Therefore, understanding the magnitude and pattern of orthopedic maxillary displacement in patients approaching skeletal maturity remains clinically relevant. Some previous studies involving patients with cleft lip and palate have reported substantial skeletal changes after Alt-RAMEC protocols [37,38]. However, because the present study included only non-cleft skeletal Class III patients, these findings cannot be directly extrapolated to cleft populations.

Several limitations should be considered when interpreting the findings of the present study. First, the retrospective design may have introduced selection bias despite the use of standardized inclusion criteria. Moreover, no untreated age- and skeletal maturity-matched Class III control group was available. Consequently, the observed positional changes in the orthopedic groups could not be distinguished definitively from the natural craniofacial growth trajectory. Second, the treatment groups were not matched for age, skeletal maturation, baseline sagittal skeletal characteristics, or observation period. This imbalance reflects the clinical indications of the three treatment modalities, as conventional RME/FM therapy is typically performed in younger growing patients, the Alt-RAMEC/2-HME protocol was applied to patients in the late-growth period, and Le Fort I osteotomy was performed in skeletally mature adults with more severe sagittal maxillary deficiency. Furthermore, post-treatment records were obtained after approximately 10 months in the RME/FM group but after only 9 weeks in the Alt-RAMEC/2-HME group, which may have resulted in different contributions of physiological growth to the observed positional changes. Because treatment outcomes were assessed at protocol-specific clinical endpoints, the different observation intervals may have contributed to the magnitude of the observed positional changes. In addition, baseline differences in sagittal skeletal severity may also have influenced the magnitude of treatment-related A-point displacement. Therefore, the observed intergroup differences may reflect both treatment-related effects and baseline patient characteristics, and the independent contribution of each factor could not be determined within the present study design. Together, these factors represent potential sources of confounding and should be considered when interpreting the intergroup comparisons. Third, although the sample size calculation supported the planned sample size for the primary outcome, it was based on the assumptions specified during the a priori study design, whereas the final analyses were performed using non-parametric methods because the assumptions of normality were not met. The relatively small number of participants in each group may also limit the generalizability of the findings. In addition, sex-specific differences in skeletal maturation and treatment response could not be evaluated because the sample size was insufficient for meaningful subgroup analyses. Fourth, post-treatment radiographs were obtained immediately after completion of the active treatment phase; therefore, the long-term stability of the observed skeletal and dentoalveolar changes could not be evaluated. Finally, the cephalometric evaluation was performed using two-dimensional lateral cephalometric radiographs, which may not fully represent the three-dimensional skeletal and dentoalveolar changes associated with maxillary expansion and protraction protocols. Furthermore, the positional changes observed in dentoalveolar landmarks could not be differentiated into true tooth movement and displacement of the surrounding alveolar process. In addition, all measurements were performed by a single investigator who was not blinded to treatment group or timepoint, which may have introduced measurement bias despite the excellent intra-observer reliability. The same investigator also performed the eligibility screening and applied the inclusion and exclusion criteria, resulting in limited independence between participant selection and outcome assessment and representing an additional potential source of bias.

Future prospective longitudinal studies with larger sample sizes, better-matched comparison groups, and three-dimensional imaging methods are needed to confirm these findings and further clarify the long-term skeletal and dentoalveolar changes associated with the Alt-RAMEC/2-HME protocol.

## 5. Conclusions

Within the limitations of this retrospective cephalometric study, sagittal A-point displacement was observed following the Alt-RAMEC/2-HME protocol in late-growth skeletal Class III patients with maxillary retrusion. However, because of the retrospective study design and the absence of an untreated age- and skeletal maturity-matched control group, the observed displacement cannot be distinguished definitively from the natural craniofacial growth trajectory. No statistically significant difference in sagittal A-point displacement was observed between the Alt-RAMEC/2-HME and conventional RME/FM groups. Greater dentoalveolar positional changes were observed in the RME/FM group, particularly in the maxillary incisor and molar regions, although these findings should be interpreted in light of the baseline differences between the study groups. Similarly, the larger sagittal A-point displacement observed in the Le Fort I osteotomy group should be interpreted as a quantitative surgical reference rather than as evidence of comparative treatment superiority.

## Figures and Tables

**Table 1:** Baseline demographic and skeletal characteristics of the study groups.

Variable	Group 1 Alt-RAMEC/2-HME (n = 14)	Group 2 RME/FM (n = 14)	Group 3 Le Fort I osteotomy (n = 14)	*p*-Value
Age, years	15.35 ± 1.01	11.40 ± 1.16	24.75 ± 1.57	<0.001
Sex, female/male	8/6	7/7	6/8	0.751
Skeletal age, years	15.25 ± 1.17	12.25 ± 1.02	NA	<0.001 †
Observation interval ‡	9 weeks	10.1 ± 1.9 months	Immediate postoperative	—
SNA	78.12 ± 1.64	78.46 ± 1.51	76.85 ± 1.72	0.028
SNB	80.34 ± 1.92	80.01 ± 1.74	80.52 ± 2.08	0.746
ANB	−2.21 ± 1.18	−1.55 ± 1.06	−3.67 ± 1.35	0.003

Values are presented as mean ± standard deviation unless otherwise indicated. Group 1: Alt-RAMEC/2-HME, alternate rapid maxillary expansion and constriction using a two-hinged maxillary expander; Group 2: RME/FM, rapid maxillary expansion followed by facemask therapy; Group 3: Le Fort I osteotomy, adult surgical reference group. NA, not applicable. *p*-values were calculated using the Kruskal–Wallis test for continuous variables and the chi-square test or Fisher’s exact test for categorical variables, as appropriate. † Skeletal age was compared only between the two orthopedic groups using the Mann–Whitney U test because hand–wrist maturation assessment was not performed in the skeletally mature Le Fort I osteotomy group. ‡ Observation interval is presented descriptively because post-treatment records were obtained at protocol-specific clinical endpoints rather than after standardized follow-up intervals; therefore, no statistical comparison was performed.

**Table 2:** Within-group comparison of skeletal and alveolar linear measurements between T0 and T1 in the study groups.

Parameter	Group 1			Group 2			Group 3		
	T0	T1	*p*	T0	T1	*p*	T0	T1	*p*
Skeletal/alveolar sagittal									
A-Y	50.44 ± 3.73	52.31 ± 4.67	0.005	48.25 ± 5.39	50.94 ± 4.33	0.002	53.87 ± 4.76	62.23 ± 4.40	0.001
B-Y	49.30 ± 8.19	48.35 ± 9.06	0.249	45.77 ± 9.01	43.96 ± 7.09	0.013	54.59 ± 6.14	53.90 ± 5.93	0.079
Pr-Y	51.79 ± 4.90	53.88 ± 5.82	0.001	48.11 ± 5.70	51.34 ± 4.69	0.001	56.69 ± 5.31	64.75 ± 4.98	0.001
Id-Y	50.85 ± 7.62	48.46 ± 7.88	0.001	47.15 ± 8.47	45.38 ± 6.38	0.019	56.26 ± 7.31	55.87 ± 6.51	0.064
Skeletal/alveolar vertical									
A-X	52.64 ± 3.85	52.63 ± 4.33	0.925	50.90 ± 4.77	50.51 ± 4.97	0.315	55.11 ± 3.09	54.56 ± 3.44	0.171
B-X	90.38 ± 6.01	92.80 ± 4.72	0.005	85.87 ± 5.95	88.71 ± 5.79	0.002	87.29 ± 12.33	85.90 ± 10.77	0.087
Pr-X	64.06 ± 5.09	64.25 ± 4.77	0.801	58.87 ± 4.83	59.64 ± 5.04	0.028	66.71 ± 3.75	65.72 ± 3.20	0.013
Id-X	85.96 ± 6.24	87.86 ± 5.60	0.048	81.12 ± 6.04	83.10 ± 6.89	0.008	82.09 ± 6.70	79.90 ± 4.47	0.012

Data are presented as mean ± standard deviation. Within-group comparisons between T0 and T1 were performed using the Wilcoxon signed-rank test. Statistical significance was set at *p* < 0.05. Abbreviations: T0, pre-treatment; T1, post-treatment; X, horizontal reference plane; Y, vertical reference plane; A, A-point; B, B-point; Pr, prosthion; Id, infradentale.

**Table 3:** Within-group comparison of dentoalveolar linear measurements between T0 and T1 in the study groups.

Parameter	Group 1			Group 2			Group 3		
	T0	T1	*p*	T0	T1	*p*	T0	T1	*p*
Dentoalveolar sagittal									
IS-Y	52.39 ± 5.97	55.04 ± 6.95	0.002	48.68 ± 6.66	53.12 ± 5.12	0.001	56.43 ± 5.60	64.89 ± 5.98	0.001
AS-Y	44.77 ± 3.51	46.54 ± 4.68	0.005	41.94 ± 5.21	44.64 ± 4.66	0.001	52.23 ± 6.94	60.21 ± 6.68	0.001
II-Y	53.66 ± 6.66	51.67 ± 6.56	0.001	50.69 ± 6.89	48.71 ± 5.62	0.003	57.60 ± 6.19	57.05 ± 5.81	0.148
AI-Y	44.66 ± 8.52	44.64 ± 9.48	0.826	40.81 ± 9.58	39.79 ± 8.12	0.221	53.01 ± 10.89	51.59 ± 11.30	0.006
UM-Y	27.65 ± 5.40	29.08 ± 5.48	0.001	25.78 ± 6.35	29.31 ± 5.25	0.001	28.15 ± 2.61	35.42 ± 4.46	0.001
UMA-Y	28.51 ± 4.10	29.76 ± 4.42	0.012	26.38 ± 4.85	29.96 ± 3.55	0.001	28.32 ± 2.09	35.57 ± 4.54	0.001
LM-Y	30.64 ± 6.77	29.01 ± 7.49	0.006	25.62 ± 7.37	25.51 ± 5.65	1.000	30.41 ± 3.55	30.75 ± 3.88	0.451
LMA-Y	21.87 ± 7.94	21.99 ± 9.12	0.624	17.41 ± 8.67	16.79 ± 6.92	0.158	27.48 ± 4.67	27.48 ± 4.42	0.730
Dentoalveolar vertical									
IS-X	75.16 ± 5.16	74.83 ± 5.57	0.593	70.53 ± 4.90	71.57 ± 4.97	0.033	75.98 ± 4.34	75.56 ± 2.36	0.777
AS-X	53.93 ± 4.62	53.90 ± 5.02	0.727	50.10 ± 4.67	50.54 ± 4.12	0.382	55.81 ± 2.62	55.09 ± 2.66	0.014
II-X	75.13 ± 5.89	76.20 ± 5.49	0.116	69.62 ± 5.61	70.68 ± 5.23	0.030	77.67 ± 4.54	75.01 ± 2.94	0.003
AI-X	92.59 ± 5.80	94.65 ± 4.55	0.010	87.40 ± 5.90	89.84 ± 5.48	0.002	88.40 ± 9.73	87.14 ± 8.76	0.014
UM-X	65.94 ± 3.92	67.32 ± 3.71	0.016	61.06 ± 4.41	62.94 ± 3.94	0.008	65.90 ± 3.22	64.99 ± 2.94	0.031
UMA-X	53.14 ± 3.51	52.64 ± 3.93	0.345	48.01 ± 4.03	49.07 ± 3.89	0.041	49.48 ± 4.60	48.92 ± 4.66	0.028
LM-X	73.83 ± 4.16	75.14 ± 3.51	0.052	67.76 ± 4.01	69.64 ± 4.38	0.003	70.91 ± 2.84	70.35 ± 3.52	0.048
LMA-X	86.51 ± 4.30	88.03 ± 3.89	0.035	79.59 ± 3.96	82.77 ± 3.21	0.003	83.76 ± 2.91	83.36 ± 4.03	0.059

Data are presented as mean ± standard deviation. Within-group comparisons between T0 and T1 were performed using the Wilcoxon signed-rank test. Statistical significance was set at *p* < 0.05. Abbreviations: T0, pre-treatment; T1, post-treatment; X, horizontal reference plane; Y, vertical reference plane; IS, upper incisor edge; AS, upper incisor apex; II, lower incisor edge; AI, lower incisor apex; UM, most mesial point of the crown contour of the maxillary first molar; UMA, apex of the mesiobuccal root of the maxillary first molar; LM, most mesial point of the crown contour of the mandibular first molar; LMA, apex of the mesial root of the mandibular first molar.

**Table 4:** Intergroup comparison of treatment-related skeletal and alveolar linear changes.

Parameter	Group 1 Mean ± SD	Group 2 Mean ± SD	Group 3 Mean ± SD	*p*	Pairwise Comparison
Skeletal/alveolar sagittal					
A-Y	1.87 ± 2.13	2.69 ± 2.05	8.36 ± 2.87	<0.001	3 > 1, 2
B-Y	−0.95 ± 2.39	−1.81 ± 2.93	−0.69 ± 1.32	0.628	—
Pr-Y	2.09 ± 2.43	3.23 ± 1.78	8.06 ± 2.77	<0.001	3 > 2 > 1
Id-Y	−2.39 ± 1.78	−1.77 ± 3.05	−0.39 ± 2.23	0.059	—
Skeletal/alveolar vertical					
A-X	−0.01 ± 1.87	−0.39 ± 1.65	−0.55 ± 1.46	0.650	—
B-X	2.42 ± 2.75	2.84 ± 2.34	−1.39 ± 3.01	<0.001	1, 2 > 3
Pr-X	0.19 ± 2.25	0.76 ± 1.10	−0.99 ± 1.65	0.008	2 > 3
Id-X	1.90 ± 3.31	1.98 ± 2.45	−2.19 ± 3.18	0.001	1, 2 > 3

Data are presented as mean ± standard deviation. Intergroup comparisons were performed using the Kruskal–Wallis test. Overall *p*-values represent unadjusted Kruskal–Wallis tests. Bonferroni-adjusted pairwise comparisons were performed using the Mann–Whitney U test when significant intergroup differences were detected. Statistical significance was set at *p* < 0.05. Abbreviations: X, horizontal reference plane; Y, vertical reference plane; A, A-point; B, B-point; Pr, prosthion; Id, infradentale.

**Table 5:** Intergroup comparison of treatment-related dentoalveolar linear changes.

Parameter	Group 1 Mean ± SD	Group 2 Mean ± SD	Group 3 Mean ± SD	*p*	Pairwise Comparison
Dentoalveolar sagittal					
IS-Y	2.64 ± 2.66	4.44 ± 2.54	8.46 ± 2.22	<0.001	3 > 2 > 1
AS-Y	1.76 ± 2.20	2.70 ± 2.19	7.98 ± 1.57	<0.001	3 > 1, 2
II-Y	−1.99 ± 1.23	−1.98 ± 1.92	−0.55 ± 1.40	0.039	1, 2 > 3
AI-Y	−0.02 ± 2.98	−1.02 ± 2.93	−1.41 ± 1.46	0.173	—
UM-Y	1.43 ± 1.55	3.53 ± 2.18	7.27 ± 2.34	<0.001	3 > 2 > 1
UMA-Y	1.26 ± 1.69	3.58 ± 2.29	7.25 ± 2.67	<0.001	3 > 2 > 1
LM-Y	−1.63 ± 1.81	−0.11 ± 2.87	0.34 ± 2.98	0.152	—
LMA-Y	0.11 ± 3.04	−0.61 ± 3.28	0.00 ± 1.36	0.827	—
Dentoalveolar vertical					
IS-X	−0.34 ± 1.66	1.04 ± 1.54	−0.42 ± 2.45	0.051	—
AS-X	−0.03 ± 1.41	0.44 ± 1.55	−0.72 ± 1.20	0.085	—
II-X	1.07 ± 2.40	1.06 ± 1.41	−2.66 ± 3.19	<0.001	1, 2 > 3
AI-X	2.06 ± 2.71	2.44 ± 1.91	−1.26 ± 2.22	<0.001	1, 2 > 3
UM-X	1.38 ± 1.93	1.89 ± 2.00	−0.91 ± 1.39	<0.001	1, 2 > 3
UMA-X	−0.50 ± 1.93	1.06 ± 1.58	−0.56 ± 1.14	0.016	2 > 1, 3
LM-X	1.31 ± 2.32	1.88 ± 1.89	−0.56 ± 0.96	0.001	1, 2 > 3
LMA-X	1.52 ± 2.27	3.19 ± 2.31	−0.39 ± 1.47	<0.001	1, 2 > 3

Data are presented as mean ± standard deviation. Intergroup comparisons were performed using the Kruskal–Wallis test. Overall *p*-values represent unadjusted Kruskal–Wallis tests. Bonferroni-adjusted pairwise comparisons were performed using the Mann–Whitney U test when significant intergroup differences were detected. Statistical significance was set at *p* < 0.05. Abbreviations: X, horizontal reference plane; Y, vertical reference plane; IS, upper incisor edge; AS, upper incisor apex; II, lower incisor edge; AI, lower incisor apex; UM, most mesial point of the crown contour of the maxillary first molar; UMA, apex of the mesiobuccal root of the maxillary first molar; LM, most mesial point of the crown contour of the mandibular first molar; LMA, apex of the mesial root of the mandibular first molar.

## Data Availability

The data presented in this study are not publicly available due to ethical and privacy restrictions, as they are derived from anonymized patient radiographic records obtained from institutional archives. Data may be made available from the corresponding author upon reasonable request and with permission of the relevant ethics committee.

## Supplementary Materials

The following supporting information can be downloaded at: https://www.mdpi.com/article/10.3390/diagnostics16162630/s1. Table S1: Within-group treatment-related changes (Δ = T1 − T0) in skeletal and alveolar measurements with corresponding 95% confidence intervals; Table S2: Within-group treatment-related changes (Δ = T1 − T0) in dentoalveolar measurements with corresponding 95% confidence intervals.

## Abbreviations

RME	Rapid Maxillary Expansion
Alt-RAMEC	Alternate Rapid Maxillary Expansion and Constriction
2-HME	2-Hinged Maxillary Expander
FM	Face Mask
PNS	Posterior Nasal Spina
