# Supplementary material for: Two-Dimensional Cephalometric Assessment of Skeletal and Dentoalveolar Changes Following Alt-RAMEC with a 2-Hinged Maxillary Expander and RME/Facemask Therapy in Skeletal Class III Patients with Maxillary Retrusion: A Retrospective Comparative Study
Source: Diagnostics (Basel). 2026 Aug 19;16(16):2630. doi: 10.3390/diagnostics16162630 (PMC13512669; doi:10.3390/diagnostics16162630)
Supplement: Supplementary file 1 [file diagnostics-16-02630-s001.zip › diagnostics-4435739-supplementary.pdf]

Table S1. Within-group treatment-related changes (Δ = T1 − T0) in skeletal and alveolar measurements with corresponding 95% confidence intervals.

Parameter	Group 1Δ Mean ± SD (95% CI)	Group 2Δ Mean ± SD (95% CI)	Group 3Δ Mean ± SD (95% CI)
**Skeletal/alveolar sagittal**			
A-Y	1.87 ± 2.13 (95% CI: 0.64–3.10)	2.69 ± 2.05 (95% CI: 1.51–3.87)	8.36 ± 2.87 (95% CI: 6.70–10.02)
B-Y	−0.95 ± 2.39 (95% CI: −2.33–0.43)	−1.81 ± 2.93 (95% CI: −3.50–−0.12)	−0.69 ± 1.32 (95% CI: −1.45–0.07)
Pr-Y	2.09 ± 2.43 (95% CI: 0.69–3.49)	3.23 ± 1.78 (95% CI: 2.20–4.26)	8.06 ± 2.77 (95% CI: 6.46–9.66)
Id-Y	−2.39 ± 1.78 (95% CI: −3.42–−1.36)	−1.77 ± 3.05 (95% CI: −3.53–−0.01)	−0.39 ± 2.23 (95% CI: −1.68–0.90)
**Skeletal/alveolar vertical**			
A-X	−0.01 ± 1.87 (95% CI: −1.09–1.07)	−0.39 ± 1.65 (95% CI: −1.34–0.56)	−0.55 ± 1.46 (95% CI: −1.39–0.29)
B-X	2.42 ± 2.75 (95% CI: 0.83–4.01)	2.84 ± 2.34 (95% CI: 1.49–4.19)	−1.39 ± 3.01 (95% CI: −3.13–0.35)
Pr-X	0.19 ± 2.25 (95% CI: −1.11–1.49)	0.76 ± 1.10 (95% CI: 0.13–1.39)	−0.99 ± 1.65 (95% CI: −1.94–−0.04)
Id-X	1.90 ± 3.31 (95% CI: −0.01–3.81)	1.98 ± 2.45 (95% CI: 0.57–3.39)	−2.19 ± 3.18 (95% CI: −4.03–−0.35)

Values represent treatment-related changes (Δ = T1 − T0) and are presented as mean ± standard deviation (SD) with corresponding 95% confidence intervals (95% CI). Abbreviations are as defined in the main manuscript.

Table S2. Within-group treatment-related changes (Δ = T1 − T0) in dentoalveolar measurements with corresponding 95% confidence intervals.

Parameter	Group 1Δ Mean ± SD (95% CI)	Group 2Δ Mean ± SD (95% CI)	Group 3Δ Mean ± SD (95% CI)
**Dentoalveolar sagittal**			
IS-Y	2.64 ± 2.66 (95% CI: 1.10–4.18)	4.44 ± 2.54 (95% CI: 2.97–5.91)	8.46 ± 2.22 (95% CI: 7.18–9.74)
AS-Y	1.76 ± 2.20 (95% CI: 0.49–3.03)	2.70 ± 2.19 (95% CI: 1.44–3.96)	7.98 ± 1.57 (95% CI: 7.07–8.89)
II-Y	−1.99 ± 1.23 (95% CI: −2.70–−1.28)	−1.98 ± 1.92 (95% CI: −3.09–−0.87)	−0.55 ± 1.40 (95% CI: −1.36–0.26)
AI-Y	−0.02 ± 2.98 (95% CI: −1.74–1.70)	−1.02 ± 2.93 (95% CI: −2.71–0.67)	−1.41 ± 1.46 (95% CI: −2.25–−0.57)
UM-Y	1.43 ± 1.55 (95% CI: 0.54–2.32)	3.53 ± 2.18 (95% CI: 2.27–4.79)	7.27 ± 2.34 (95% CI: 5.92–8.62)
UMA-Y	1.26 ± 1.69 (95% CI: 0.28–2.24)	3.58 ± 2.29 (95% CI: 2.26–4.90)	7.25 ± 2.67 (95% CI: 5.71–8.79)
LM-Y	−1.63 ± 1.81 (95% CI: −2.68–−0.58)	−0.11 ± 2.87 (95% CI: −1.77–1.55)	0.34 ± 2.98 (95% CI: −1.38–2.06)
LMA-Y	0.11 ± 3.04	−0.61 ± 3.28	0.00 ± 1.36

	(95% CI: −1.65–1.87)	(95% CI: −2.50–1.28)	(95% CI: −0.78–0.78)
**Dentoalveolar vertical**			
IS-X	−0.34 ± 1.66<br>(95% CI: −1.30–0.62)	1.04 ± 1.54<br>(95% CI: 0.15–1.93)	−0.42 ± 2.45<br>(95% CI: −1.83–0.99)
AS-X	−0.03 ± 1.41<br>(95% CI: −0.84–0.78)	0.44 ± 1.55<br>(95% CI: −0.45–1.33)	−0.72 ± 1.20<br>(95% CI: −1.41–−0.03)
II-X	1.07 ± 2.40<br>(95% CI: −0.32–2.46)	1.06 ± 1.41<br>(95% CI: 0.25–1.87)	−2.66 ± 3.19<br>(95% CI: −4.50–−0.82)
AI-X	2.06 ± 2.71<br>(95% CI: 0.50–3.62)	2.44 ± 1.91<br>(95% CI: 1.34–3.54)	−1.26 ± 2.22<br>(95% CI: −2.54–0.02)
UM-X	1.38 ± 1.93<br>(95% CI: 0.27–2.49)	1.89 ± 2.00<br>(95% CI: 0.74–3.04)	−0.91 ± 1.39<br>(95% CI: −1.71–−0.11)
UMA-X	−0.50 ± 1.93<br>(95% CI: −1.61–0.61)	1.06 ± 1.58<br>(95% CI: 0.15–1.97)	−0.56 ± 1.14<br>(95% CI: −1.22–0.10)
LM-X	1.31 ± 2.32<br>(95% CI: −0.03–2.65)	1.88 ± 1.89<br>(95% CI: 0.79–2.97)	−0.56 ± 0.96<br>(95% CI: −1.11–−0.01)
LMA-X	1.52 ± 2.27<br>(95% CI: 0.21–2.83)	3.19 ± 2.31<br>(95% CI: 1.86–4.52)	−0.39 ± 1.47<br>(95% CI: −1.24–0.46)

Values represent treatment-related changes (Δ = T1 − T0) and are presented as mean ± standard deviation (SD) with corresponding 95% confidence intervals (95% CI). Abbreviations are as defined in the main manuscript.
